# A Review on the Use of Grid-Based Boltzmann Equation Solvers for Dose Calculation in External Photon Beam Treatment Planning

**DOI:** 10.1155/2013/692874

**Published:** 2013-08-27

**Authors:** Monica W. K. Kan, Peter K. N. Yu, Lucullus H. T. Leung

**Affiliations:** ^1^Department of Oncology, Princess Margaret Hospital, Hong Kong; ^2^Department of Physics and Materials Science, City University of Hong Kong, Tat Chee Avenue, Kowloon Tong, Hong Kong

## Abstract

Deterministic linear Boltzmann transport equation (D-LBTE) solvers have recently been developed, and one of the latest available software codes, Acuros XB, has been implemented in a commercial treatment planning system for radiotherapy photon beam dose calculation. One of the major limitations of most commercially available model-based algorithms for photon dose calculation is the ability to account for the effect of electron transport. This induces some errors in patient dose calculations, especially near heterogeneous interfaces between low and high density media such as tissue/lung interfaces. D-LBTE solvers have a high potential of producing accurate dose distributions in and near heterogeneous media in the human body. Extensive previous investigations have proved that D-LBTE solvers were able to produce comparable dose calculation accuracy as Monte Carlo methods with a reasonable speed good enough for clinical use. The current paper reviews the dosimetric evaluations of D-LBTE solvers for external beam photon radiotherapy. This content summarizes and discusses dosimetric validations for D-LBTE solvers in both homogeneous and heterogeneous media under different circumstances and also the clinical impact on various diseases due to the conversion of dose calculation from a conventional convolution/superposition algorithm to a recently released D-LBTE solver.

## 1. Introduction

Highly conformal photon dose distributions in various treatment sites can be achieved using different techniques of multileaf collimator-based intensity modulated radiotherapy, including static intensity modulated radiotherapy (IMRT) and volumetric modulated arc therapy (VMAT). Radiotherapy using intensity modulated techniques improves the possibility to escalate the target dose and minimize doses to critical organs when compared to three-dimensional conformal radiotherapy [1–11]. The use of IMRT or VMAT in patients usually involves many small field segments, some of which might pass through regions of low and high density media such as lung, air, and bone, depending on the location of the tumor and the surrounding normal tissues. One issue that affects the dose calculation accuracy in highly conformal planning is the ability of the algorithm to correctly account for the effects of radiation transport with the presence of heterogeneous medium.

Correction-based algorithms implemented in commercially available clinical treatment planning system include the pencil beam algorithm (PBC), collapsed cone convolution algorithm (CCC), and the analytical anisotropic algorithm (AAA). For PBC, it assumes that any collimated photon beam incident on the patient is composed of a large number of infinitely narrow pencil beams of photons. The total dose is calculated by superposition of pencil beam dose kernels at each point in space around the incident beam derived from Monte Carlo simulations. The effects of tissue variations and patient contour are usually modeled based on equivalent path length methods or the modified Batho correction method [12–14]. More advanced superposition/convolution methods such as AAA and CCC are able to incorporate electron and secondary photon transport in an approximate way for dose calculations in a heterogeneous medium. These methods use the superposition of the Monte Carlo derived dose kernels of both primary and scatter components to obtain doses in voxels of the irradiated volume. To account for the presence of inhomogeneities, simple density scaling of the kernels is applied so that the secondary electron transport is only modeled macroscopically. Both AAA and CCC were proved to produce inaccurate dose distribution in media with complex heterogeneities in certain circumstances [14–18].

The Monte Carlo (MC) methods have been considered the most accurate methods for radiotherapy treatment planning dose calculation. They are statistical simulation methods based on random sampling. They solve the radiation transport problem stochastically by simulating the tracks of a sufficiently large number of individual particles using the random number generated probability distribution governing the individual physical processes. They are therefore capable of accurately computing the radiation dose in media under almost all circumstances [19, 20]. However, the computation time required may still limit the use of MC methods for complex intensity modulated techniques in the clinical environment. 

The desire to develop a fast alternative dose calculation method with comparable accuracy to MC methods has led to the exploration of deterministic solutions to the coupled system of linear Boltzmann transport equations (LBTE) [21–26]. It was first demonstrated using the prototype software, Attila, which was a general purpose grid-based Boltzmann solver code. It was followed by Acuros developed by Transpire, Inc. (Gig Harbor, WA, USA), specially designed for radiotherapy dose calculations. Recently, a version of deterministic LBTE solver, namely, Acuros XB (AXB), has been developed and implemented in the Eclipse treatment planning system (Varian Medical Systems, Palo Alto, CA, USA). The LBTE is the governing equation that describes the macroscopic behavior of ionizing particles as they travel through and interact with matter. The electron angular fluence is first obtained by solving the LBTE, and then the dose can be generated by using the macroscopic electron energy deposition cross-sections and the density of the materials. With sufficient refinement without using any approximation, that is, if an MC algorithm simulates an infinite number of particles and a deterministic LBTE (D-LBTE) solver discretizes the variables such as space and energy into infinitely small grids, both approaches will converge on the same solution. The achievable accuracy of both approaches is equivalent and is limited only by uncertainties in the particle interaction data and uncertainties related to the transported radiation fields. Extensive efforts have been made by several investigators to validate the accuracy of D-LBTE solvers in different circumstances by comparison against MC and by experimental verification against measurements. Performed validations ranged from using a simple geometric phantom with simple fields to a complex humanoid phantom with multiple intensity modulated fields. Most studies reported that D-LBTE solvers were capable of producing comparable accuracy as MC methods and either equivalent or better accuracy than superposition/convolution algorithms [22–35]. Dosimetric impact on different media of various clinical sites due to the conversion of the currently used model-based algorithms to the newly implemented D-LBTE solvers was also investigated by several authors [32, 36–38]. This paper summarizes and discusses the findings of the most recent dosimetric evaluation for D-LBTE solvers in various treatment sites. 

## 2. The Deterministic LBTE Solvers

More detailed description of the D-LBTE solvers can be found in the literature [21–26]. Only a summary is reported here. 

The time-independent three-dimensional (3D) system of the coupled LBTE is solved to determine the energy deposition of photon and electron transport:

(1) $$\begin{array}{c} \hat{\Omega }\cdot \overset{⃑}{\nabla }\Phi^{\gamma }+\sigma_{\;}^{\gamma }\Phi^{\gamma }=q^{\gamma \gamma }+q^{\gamma }, \end{array}$$

(2) Ω^·∇⃑Φe+σ  eΦe−∂∂E(SRΦe)=qee+qγe+qe,      r⃑∈V,  Ω^∈4π,  E>0,

where Φ^*γ*^ and Φ^*e*^ are the photon and electron angular fluence, respectively, *σ*_  _^*γ*^ and *σ*_  _^*e*^ are the macroscopic photon and electron total interaction cross-sections for all materials in volume *V* and energies, respectively, *q* ^*γγ*^, *q* ^*γe*^, and *q* ^*ee*^ represent the photon scattering source generated from photon interaction, electron scattering source generated from photon interaction, and electron scattering source generated from electron interactions everywhere in *V* for all angles and energies, respectively, *q* ^*γ*^ and *q* ^*e*^ represent the external photon and electron source from the treatment head, respectively, $\overset{⃑}{r}$ is the spatial position vector, *E* is the energy, $\hat{\Omega }$ is the unit vector denoting particle direction, and $\overset{⃑}{\nabla \;}$ is referred to as the “streaming operator” which may be interpreted as the number of particles flowing into a volume *dV*, minus the number of particles flowing out of *dV* for particles travelling in a direction *d*Ω about Ω with energy *E* about *dE*. The second terms on the left-hand side of (1) and (2) are the “collision operators,” which may be thought of as the number of particles removed from the volume by absorption or scattering. Equation (2) is the Boltzmann Fokker-Plank transport equation, which is solved for the electron transport. The third term on the left-hand side of (2) represents the continuous slowing down operator, where *S*_*R*_ is the restricted plus collisional radiative stopping power. Equations (1) and (2) are usually solved through discretization in space, angle, and energy. Energy discretization is achieved with the standard multigroup method. Space discretization can be achieved with a variably sized Cartesian adaptive mesh refinement technique (used by AXB) or by using a high-order Galerkin-based linear discontinuous finite-element method to solve the multigroup discrete ordinates equations on fully unstructured tetrahedral elements (used by Attila). For the former technique, the mesh is limited to refinement in factors of 2 or smaller in any direction. This allows for the use of finer resolution in higher dose and high dose gradient regions. Angle discretization for fluences and scattering sources is achieved with the standard ordinates method, where the quadrature order is adaptive by the energy group. The photon angular fluence of (1) is the summation of uncollided (primary photon without interaction with matter) and collided fluence components (photons produced or scattered by photon interactions in the patient), where the latter is discretized using a linear discontinuous finite-element method, providing a linear solution variation throughout each element, with discontinuities permitted across element faces. After solving the electron angular fluence, the dose in any grid voxel, *i*, is calculated as follows:

(3) $$\begin{array}{c} D_{i}=\int_{0}^{1}dE\int_{4\pi }^{}\frac{d\hat{\Omega }\left(\sigma_{\text{ED}}^{e}\left(\overset{⃑}{r},E\right)\right)}{\rho \left(\overset{⃑}{r}\right)}\Phi^{e}\left(\overset{⃑}{r},E,\hat{\Omega }\right), \end{array}$$

where *σ*_ED_^*e*^ is the macroscopic energy deposition cross-section and *ρ* is the material density of the local voxel.

Similar to the MC methods, D-LBTE solvers also use energy cut-offs for electrons and photons. A particle is assumed to deposit all of its energy locally below the cut-off energy. For example, AXB uses an electron cut-off energy of 500 keV and a photon cut-off energy of 10 keV. Assumptions similar to those used in some MC methods are also applied to (1) and (2) of D-LBTE solvers. It is assumed that both secondary charged particles produced by pair production are electrons, not one electron plus one positron. It is also assumed that photons produce electrons, but electrons do not produce photons. The energy from photons produced by the electrons is assumed to be deposited locally. For (2), it is assumed that the Fokker-Planck operator is used for “soft” interactions leading to small-energy losses. Catastrophic interactions leading to large energy losses are represented with the standard Boltzmann scattering.

Both MC methods and D-LBTE solvers produce errors. MC methods produce stochastic errors when an insufficient number of particle histories is followed. LBTE solvers produce systematic errors due to finite discretization resolution in space, angle, and energy. Better accuracy always requires longer computation time. In addition, the achievable accuracy of MC and D-LBTE solver is limited by uncertainties in particle interaction data, patient geometry, and composition of the radiation field being modeled.

Similar to some MC methods, two options of dose reporting modes, that is, dose-to-water, *D*_*w*_, and dose-to-medium, *D*_*m*_, are usually provided in D-LBTE solvers. Both options calculate dose considering the elemental composition of each material in which particles are transported. The difference between them is mainly in the postprocessing step, in which *D*_*w*_ is obtained by rescaling *D*_*m*_ using the stopping power ratio of water to medium.

## 3. Validation in Homogeneous Water

It is important to validate a new dose calculation algorithm in basic geometrical conditions such as that in homogeneous water before going ahead for more complicated ones. The information regarding the accuracy in simple cases is important to identify the sources of errors or uncertainties in more complicated geometries. Fogliata et al. performed a comprehensive assessment of AXB in Eclipse to model photon beams of low and high energy in homogeneous water with simple geometries [26]. They also included “flattening filter free” (FFF) beams from the Varian TrueBeam machine. The use of an FFF beam significantly increases the dose rate and therefore reduces the delivery time of a treatment machine. Due to the removal of flattening filter, the physical aspects of FFF beams are different from those of conventional flattened ones, including forward peaked intensity profiles in the middle instead of uniform flat profiles across the fields, steeper dose fall-off of percentage depth doses in the exponential region, less variation of off-axis beam hardening, lower mean energy, less photon head scatter, and higher surface dose. For conventional flattened beams, the performance of AXB was determined by comparison of calculated data against measured data in water for open and wedged fields. For FFF beams, the verification tests were performed for open fields only. The overall accuracy was found to be within 1% for open beams and 2% for mechanical wedges. 

Testing the performance of AXB using open fields in homogeneous water was also performed by several other investigators [27, 28, 32]. Doses calculated using AXB were compared to measured/golden beam data, data calculated using AAA and CCC, as well as MC simulated data using different field sizes for different energy beams. Output factors, percentage depth doses (PDD), and lateral dose profiles at various depths were examined. In general, the agreement between the calculated data generated by the various models and the measured/golden beam data were found to be better than or close to 2%, with slightly larger discrepancies found in the build-up and penumbra regions. The calculated penumbral widths were usually found to be slightly smaller than the measured ones. 

In homogeneous water, comparable performance was found between AXB and AAA/CCC. This was expected as most commercially available correction-based algorithms, and radiation transport algorithms were capable of accurately predicting the photon beam dose distribution in homogenous water. The discrepancies between calculated data and measured data were mostly limited by the precision and spatial resolution of the beam measurement devices used, especially in regions of high dose gradient. For example, the use of ion chamber with finite size for measuring dose profiles would broaden the penumbra width. 

## 4. Verification with Inhomogeneous Simple Geometric Phantom Using Single Open Fields

Several investigations have been performed to examine the accuracy of several different D-LBTE solvers for predicting the dose distribution in heterogeneous simple geometric phantoms using single fields of different photon energies [22, 25, 27–31]. The media of interest included soft tissue, normal lung, light lung, air, bone, aluminium, stainless steel, and titanium alloy. Most of the verifications were performed by benchmarking against the dose distributions calculated by MC methods.  Table 1 summarizes the methods, phantom geometries, beam configurations, and comparison results between MC and D-LBTE solvers of some previous investigations. In general, good agreement was found between D-LBTE solvers and MC, with discrepancies of better than or equal to 2% in most cases. Verification using AXB of version 10 showed that there were slightly larger discrepancies of up to about 4 to 6% found in the presence of very low density media such as light lung or air, especially at/near the interface in the secondary build-up region when small fields were used [27, 30]. The accuracy of D-LBTE solvers depends on the material assignment and the level of sampling the structure voxels to the calculation grid. Fogliata et al. showed that the version 11 of AXB gave better agreement with MC when predicting doses in the presence of air than the version 10.0 of AXB, which was due to the inclusion of air material assignment (air material was not included in version 10.0) and the provision of better resampling process of the structure voxels to the calculation grid [29].

Some of these studies also compared the accuracy of AXB with AAA [29–31], one of which performed the comparison with CCC as well [28]. All of them observed considerably larger differences between AAA and MC than those between AXB and MC in the presence of lung, air, and very high density objects especially near the interfaces. It was found that AXB could improve the dose prediction accuracy over both AAA and CCC in the presence of heterogeneities. It should also be noted that the depth dose profile data presented by Han et al. showed that CCC produced slightly better agreement with MC than AAA in both lung and bone regions [28].

## 5. Verification Using Multiple Clinical Setup Fields with Humanoid Geometry

### 5.1. Verification by Comparison with Monte Carlo Simulation

Some investigations were performed to examine the accuracy of D-LBTE solvers by comparison against MC methods for clinical setup fields [24, 25]. One study compared the dose distributions from one prostate and one head-and-neck clinical treatment plans calculated by Attila to those calculated by MC using the DOSXYZnrc program. Both plans were generated using the CT image data set of the real patients using multiple coplanar open fields. 3D gamma evaluation showed that 98.1% and 98.5% of the voxels passed the 3%/3 mm criterion for the prostate case and the head and neck case, respectively.

Another study compared the dose distributions from a tangential breast treatment plan calculated by Acuros (Transpire, Inc.) to those calculated by MC using the DOSXYZnrc program. The plan was generated on an anthropomorphic phantom with two tangential fields using a field-in-field technique. Field shapes were defined by a multileaf collimator using both 6 and 18 MV beams. The 3D gamma evaluation showed that the dose agreement was up to 98.7% for the 2%/1 mm criterion and reached 99.9% for the 2%/2 mm criterion. The differences were mostly found in the air external to the patient and in the lateral penumbra on the inside edge of the fields. 

In general, both studies showed excellent agreement between D-LBTE solvers and MC in all regions including those near heterogeneity and with the use of small fields. These studies indicated that D-LBTE solvers were able to produce similar accuracy as MC methods for complicated geometries. However, the achievable accuracy of MC approach was also limited by uncertainties of the particle interaction data, the geometry and composition of the field being modeled, and other approximations made in radiation transport. Comprehensive validations of D-LBTE solvers should also cover comparisons against experimental measurements. Treatment plans with more complex intensity modulated fields, such as IMRT and VMAT, were not included in these studies.

### 5.2. Verification by Comparison against Measurements

Verifications of AXB against measurements using IMRT and VMAT plans for various diseases were reported [30, 32–35]. Humanoid phantoms used include the Radiological Physics Center (RPC) phantoms, the anthropomorphic phantom (the RANDO phantom, The Phantom Laboratory, Salem, NY, USA), and the CIRS Thorax Phantom (CIRS, VA, USA).  Table 2 summarizes some of the details including methods and results of each verification study. Regarding verification using thermoluminescence dosimeters (TLDs), all the calculated data matched with the measured data are within 5%, with an average discrepancy of about 2 to 3%. The positions of measurement included those inside the heterogeneous medium and near/at the interfaces. 

For the gamma analysis using EBT films, the passing rate of the 3%/3 mm criterion met the recommendation (should be >90%) set by TG 119 for the studies performed in the nasopharyngeal region and the lung, where heterogeneities exist. However, the one performed using the RPC head and neck phantom, where only tissue equivalent material was involved, could only produce a passing rate of 88% for the 5%/3 mm criterion [33]. The inferior results reported might be due to the larger uncertainty of the film registration method during analysis.

All experimental validations listed also compared the accuracy between AXB and AAA. The accuracy of both when compared to TLD measurement was quite comparable except for the investigation using intensity modulated stereotactic radiotherapy (IMSRT) in locally persistent nasopharyngeal. For the IMSRT cases, AXB demonstrated better accuracy near air/tissue interfaces when compared with AAA. This might be due to the very small field segments used in IMSRT cases with the presence of air cavities. For validations performed with films, the accuracy of AXB was in general shown to be slightly better than that of AAA. When compared to TLD, films could measure a much larger number of points in a single measurement and provided better spatial resolution. This might be the reason why films could better distinguish between the accuracies of AAA and AXB even when the difference was small.

## 6. Dose in Medium against Dose in Water

For external photon beam radiation therapy planning, the input data used for most conventional correction/model-based dose algorithms are dose distributions and beam parameters measured in water. They usually report patient dose in terms of the absorbed dose to water (*D*_*w*_) using variable electron density. On the other hand, LBTE solvers calculate the energy deposition considering radiation particle transport in different media and therefore report dose directly to patient medium (*D*_*m*_). According to the recommendation from the American Association of Physicists in Medicine (AAPM) Task Group 105, MC results should allow conversion between *D*_*m*_ and *D*_*w*_, based on the Bragg-Gray cavity theory, either during or after the MC simulation. This recommendation also applies to all other deterministic algorithms that are able to report *D*_*m*_ accurately for plan evaluation [39]. *D*_*m*_ calculated by LBTE solvers can be converted to *D*_*w*_ using the Bragg-Gray cavity theory by

(4) $$\begin{array}{c} D_{w}=D_{m}{\left(\frac{\bar{S}}{\rho }\right)}_{m}^{w}, \end{array}$$

where ${\left(\bar{S}/\rho \right)}_{m}^{w}$ is the unrestricted water to medium mass collision stopping power ratio averaged over the energy spectra of primary electrons at the point of interest. It has been recently debated whether the *D*_*m*_ dose inherently predicted by MC methods needs to be converted to *D*_*w*_. There are certain arguments between using *D*_*m*_ and *D*_*w*_ for radiotherapy treatment planning in the clinical environment. Those supporting the use of *D*_*w*_ argued that (1) therapeutic and normal tissue tolerance doses determined from clinical trials were based on *D*_*w*_ as photon dose measurements and calculations were historically reported in terms of *D*_*w*_, (2) calibration of treatment machines were performed according to recognized dosimetry protocols in terms of the absorbed dose to water, and (3) tumor cells embedded within any medium such as bone were more water-like than medium-like. Those supporting the use of *D*_*m*_ argued that (1) the dose to the tissues of interest was the quantity inherently computed by radiation transport dose algorithms and therefore was more clinical relevant and (2) the conversion of *D*_*m*_ back to *D*_*w*_ might induce additional uncertainty to the final calculated dose. 

**Figure 1:**
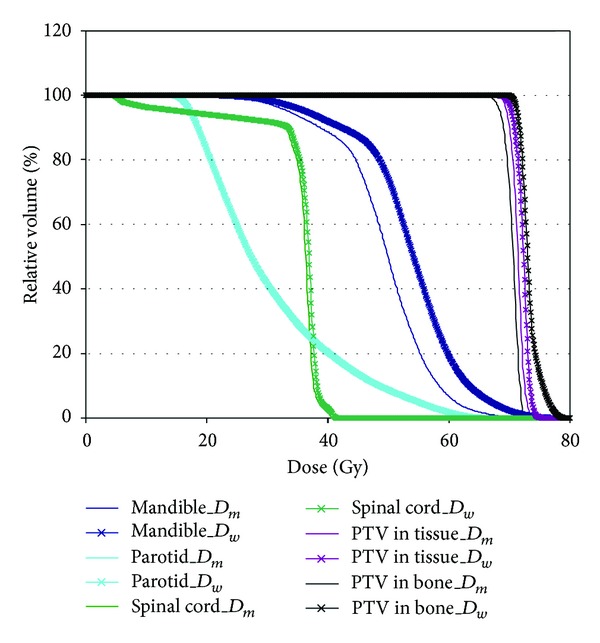
DVH curves for different OAR and PTV components generated by AXB with both *D*_*m*_ and *D*_*w*_ calculation options for a typical VMAT plan of an NPC patient.

Several studies proved that the difference between using *D*_*w*_ and *D*_*m*_ for predicting photon dose distribution mainly occurred in higher density materials such as the cortical bone. The dose discrepancy could be up to 15% due to the large difference between the stopping powers of water and these higher-density materials. For soft tissues and lung, the dose discrepancy was only about 1 to 2% [33, 35, 40]. An investigation by Dogan et al. based on the MC method found that converting *D*_*m*_ to *D*_*w*_ in IMRT treatment plans introduced a discrepancy in target and critical structure of up to 5.8% for head and neck cases and up to 8.0% for prostate cases when bony structures were involved [41]. Kan et al. also observed that AXB using *D*_*w*_ calculated up to 4% higher mean doses for the bony structure in planning target volume (PTV) when compared to *D*_*m*_ in IMRT and VMAT plans of NPC cases [34].  Figure 1 shows the difference in dose volume histograms (DVHs) between *D*_*m*_ and *D*_*w*_ for different organs at risk (OAR) and PTV components (both bone and soft tissues). They were generated by AXB using both *D*_*m*_ and *D*_*w*_ for a typical VMAT plan of an NPC case. It can be seen from the DVH curves that larger dose differences were found between *D*_*m*_ and *D*_*w*_ in organs with bony structures such as mandible than those with soft tissue such as parotids.

Previous studies using Monte Carlo and AXB calculations proved that conventional model based algorithms predicted dose distributions in bone that were closer to *D*_*m*_ distributions than to *D*_*w*_ distributions [34, 42]. It is therefore better to use *D*_*m*_ for consistency with previous radiation therapy experience.

## 7. Dosimetric Impact in Clinical Cases

Various studies were performed to assess the dosimetric impact of using AXB instead of AAA for dose calculations in different clinical cases, including lung cancer, breast cancer, and nasopharyngeal carcinomas [36–38]. AXB calculations for these investigations were all performed using the *D*_*m*_ option, so that the capability of the algorithm to distinguish between different elemental compositions in the human body could be assessed. The grid resolution for dose calculation selected was 2.5 mm. In order to evaluate the dose differences between the two algorithms due to the issue of tissue heterogeneity, the PTV were divided into components of different densities and compositions during dose analysis.

### 7.1. Lung Cancer

The clinical dosimetric impact for advanced non-small-cell lung cancer was assessed using three different techniques: three-dimensional conformal radiotherapy, IMRT, and RapidArc (the name of the VMAT system from Varian Medical Systems Inc., Palo Alto, CA, USA) at both 6 and 15 MV [36]. The PTVs were split into two components, namely, PTV in soft tissue and PTV in lung. The dose prescription was 66 Gy at 2 Gy per fraction to the mean target dose for each planning technique. The results demonstrated that AXB predicted up to 1.7% and 1.2% lower mean target doses in soft tissue for 6 MV and 15 MV beams, respectively, and up to 1.2% higher and 2.0% lower mean target doses in lung for 6 MV and 15 MV beams, respectively. In general, AAA overestimated the doses to most PTV components, except for PTV in lung when using IMRT at 6 MV, where the opposite trend was observed. AXB predicted up to 3% lower mean doses to OAR. The observed trend was similar for different treatment techniques.

### 7.2. Breast Cancer

The dosimetric impact for breast cancer was assessed using the opposing tangential field setting technique at 6 MV [37]. Doses in organs were analyzed using patient datasets scanned under two different breathing conditions, free breathing (FB, representing higher lung density), and deep inspiration (DI, representing lower lung density). The target breast was split into components in muscle and in adipose tissue. It was observed that AAA predicted 1.6% higher doses for the muscle than AXB (version 11). The difference in doses predicted by both algorithms to the adipose tissue was negligible. AAA was found to predict up to 0.5% and 1.5% higher doses than using version 11 of AXB in the lung region within the tangential field for FB and DI, respectively. The authors comparing between versions 10 and 11 of AXB found negligible differences in the predicted doses for tissue and normal lung. However, they observed that, for the lower density lung in the condition of DI, version 11 of AXB predicted an average of 1.3% higher dose than version 10. This was mainly due to the more accurate dose calculation of version 11 for very low density lung achieved by including the low density air in the material list.

### 7.3. Nasopharyngeal Carcinomas

The dosimetric impact for NPC was assessed using IMRT and RapidArc at 6 MV due to the use of AXB version 10 compared to AAA [38]. The PTVs with multiple prescriptions were separated into components in bone, air, and tissue. AAA was found to predict about 1% higher mean doses to the PTVs in tissue, 2% higher doses to the PTVs in bone, and 1% lower doses to the PTVs in air. AAA also predicted up to 3% higher doses to most serial organs. It should be noted that AAA predicted up to 4% higher minimum doses to the PTVs in bone, where the gross tumor volume was located. 

On the whole, the various investigations for different treatment sites listed above demonstrated that in general AAA predicted higher doses to PTV and OAR, when compared with AXB. The overestimation by AAA was mostly within 2% in soft tissues such as muscle and lung and could be up to 4% in bone. 

## 8. Discussions

Various studies showed that D-LBTE solvers were able to produce satisfactory dose calculation accuracy in the presence of heterogeneous media, even at and near interfaces of different material densities [22–35]. They were proved to produce equivalent accuracy to MC methods and better accuracy than convolution/superposition algorithms. These results are expected as D-LBTE methods model the radiation transport process in a similar manner as MC methods. There is still room for improvement in the latest version of clinically available AXB regarding accuracy in physical material assignment and calculation speed. For example, one of the limitations of AXB is the restricted material assignment range. If the CT dataset of a high density object contains HU values corresponding to a mass density greater than 3.0 g/cm^3^, it is required to contain all voxels in a contoured structure with manual assignment of mass density. That means the mass density of the high density object must be known for accurate dose calculations. The validation of AXB by Lloyd and Ansbacher proved that it was able to predict the back-scatter and lateral-scatter dose perturbations accurately adjacent to very higher density objects (with density in the range from 4.0 to 8.0 g/cm^3^) [31]. However, in reality, this would be difficult for real patient planning due to the misinterpretation of HU values of high density implants introduced by shadow artifacts in CT images. 

When compared to MC methods, the use of D-LBTE solvers might result in relatively shorter calculation time as explicit modeling of a large number of particle interactions is not required. Previous studies observed that the earlier D-LBTE code, Attila, performed dose calculations faster than the general purpose of MC method such as EGS4 or the EGSnrc by an order of magnitude for both external beam and brachytherapy planning [22, 24]. Acuros, which was optimized for use in radiotherapy planning, was reported to perform roughly an order of magnitude faster than Attila for various clinical cases [25]. Furthermore, the latest version of D-LBTE method, AXB, was reported to produce 3 to 4 times faster speed for VMAT planning compared to AAA [36]. The above evidence indicates that D-LBTE methods can be a fast and accurate alternative to MC methods. However, it is in fact difficult to perform direct comparison of the speed between MC and D-LBTE solvers as it depends on the hardware and the efficiency of the coding used. The computation time of D-LBTE solvers might be further reduced in the future by implementation on graphical processing units and additional refinements. On the other hand, fast MC codes have been developed to improve the speed of dose calculation for clinical use. Examples include the Voxel-based Monte Carlo (VMC, VMC++), Macro Monte Carlo, Dose Planning Method (DPM), and MCDOSE [43–50]. Continuous development of more efficient MC codes in the future may compete with currently commercial available D-LBTE methods in terms of both accuracy and speed. 

Although D-LBTE solvers were proved to be more accurate than convolution/superposition algorithms, significant differences were mainly confined to certain extreme conditions. These mainly include doses near heterogeneous interfaces when using single or multiple small fields. Up to 8 to 10% higher doses near interfaces were predicted by AAA compared with AXB when stereotactic small fields were used in the presence of air cavity [30]. Smaller differences were found when using IMRT and VMAT setup fields. Several experimental verifications showed comparable dose accuracy between AXB and AAA in soft tissues within complex heterogeneous geometries for clinical intensity modulated fields [33–35]. The studies assessing the dosimetric impact of using AXB on various clinical sites also showed only about 1 to 2% lower means doses in all soft tissues predicted by AXB compared to AAA [36–38]. Slightly larger differences of about 4% were found in bony structures due to the fact that AXB reported dose to medium as default while AAA reported dose to water as default. Most of these comparison studies were confined between AAA and AXB, as both of them are implemented in the same treatment planning system. Comparison between AXB with other convolution/superposition methods such as CCC for various clinical sites is not reported. From the single field study performed by Han et al. [28] in simple heterogeneous geometry, it can be predicted that CCC may produce a closer dose distribution to AXB than AAA for clinical multiple setup fields. It is because CCC predicts more accurate doses near heterogeneous interfaces than AAA for single fields, and, like AXB, it reports dose to medium as default. 

Most dosimetric studies mentioned above indicated that AAA slightly overestimated the doses to target volumes compared to AXB. If D-LBTE methods are used instead of model-based algorithms for treatment planning, it is very likely that more doses will be given to the target volumes provided that the prescribed doses by oncologists remain unchanged. Whether such conversion will bring actual clinical impact to the patients such as improvement in tumor control probability for various clinical sites requires further investigation. 

## 9. Conclusions

On the whole, grid-based D-LBTE solvers were evaluated by extensive investigations to be accurate and valuable dose calculation methods for photon beam radiotherapy treatments involving heterogeneous materials. They were proved to produce doses in good agreement with MC methods and measurements in different clinical sites using techniques ranging from relatively simple to very complex intensity modulated treatment. The use of D-LBTE solvers is highly recommended for cases with heterogeneities. However, users must be aware of the dosimetric impact on various treatment sites due to the conversion from using model-based algorithms to D-LBTE solvers.

## Figures and Tables

**Table 1:** A summary describing information of some previous investigations for the accuracy of D-LBTE solvers in predicting the doses in heterogeneous simple geometric phantoms using single open fields.

Published investigations	Gifford et al. 2006 [22]	Vassiliev et al. 2010 [25]	Bush et al. 2011 [27]	Han et al. 2011 [28]	Kan et al. 2012 [30]	Lloyd and Ansbacher 2013 [31]
Beam energy	18 MV	6 and 18 MV	6 and 18 MV	6 and 18 MV	6 MV	6 and 18 MV

Field sizes	1.5 × 1.5 cm^2^	2.5 × 2.5 cm^2^ 5.0 × 5.0 cm^2^ 10.0 × 10.0 cm^2^	4.0 × 4.0 cm^2^ 10.0 × 10.0 cm^2^ 15.0 × 10.0 cm^2^	2.5 × 2.5 cm^2^ 5.0 × 5.0 cm^2^ 10.0 × 10.0 cm^2^	2.0 × 2.0 cm^2^ 3.0 × 3.0 cm^2^ 5.0 × 5.0 cm^2^	10.0 × 10.0 cm^2^

Phantom(s) geometry	One multilayer phantom: water (0–3 cm), aluminium, Al (3–5 cm), lung (5–12 cm), water (12–30 cm)	One multilayer phantom: water (0–3 cm), bone (3–5 cm), lung (5–12 cm), water (12–30 cm)	Two phantoms: (i) one with a single insert of normal lung, light lung, or air in water, (ii) a bone/lung phantom with several disk-shaped bony structures	One multilayer phantom: water (0–3 cm), bone (3–5 cm), lung (5–12 cm), water (12–30 cm)	30.0 × 30.0 × 30.0 cm^3^ of water containing 5.0 × 5.0 × 30.0 cm^3^ of air	20.0 × 20.0 × 20.0 cm^3^ of muscle cube containing 2.0 × 2.0 × 18.0 cm^3^ of stainless steel or titanium alloy

Monte carlo simulation	EGS4/Presta, 0.3% statistical uncertainty, resolution: 0.5 × 0.5 × 0.2 cm^3^ voxels	DOSXYZnrc, <0.1% statistical uncertainty, resolution: 0.2 × 0.2 × 0.2 cm^3^ voxels, 0.1 cm laterally in penumbra region	DOSXYZnrc ~1% statistical uncertainty in media except up to 4.5% in air, resolution: 0.25 × 0.25 × 0.25 cm^3^ voxels	DOSXYZnrc, <1% statistical uncertainty, resolution: 0.2 × 0.2 × 0.2 cm^3^ voxels for most volume, 0.1 × 0.1 × 0.2 cm^3^ near water/bone and bone/lung interfaces	EGS4/Presta, 2.0% statistical uncertainty, resolution: 1/10 of field dimensions with 0.2 mm bin thickness	DOSXYZnrc, ~1% statistical uncertainty, resolution: 0.2 × 0.2 × 0.2 cm^3^ voxels

D-LBTE solver	Attila code	Acuros (Transpire, Inc.)	AXB of version 10	AXB of version 10	AXB of version 10	AXB of version 11

Dose distribution examined	PDD	PDD and lateral profiles	PDD and lateral profiles	PDD, lateral profiles, and 3D gamma evaluation	PDD	PDD and lateral profiles

Difference between D-LBTE solver and Monte Carlo simulation	Average discrepancy is 1.4%, with 2.2% maximum discrepancy observed at water/Al interface	For 6 MV, max. discrepancy < 1.5%, with DTA < 0.7 mm in the build-up region. For 18 MV, max. discrepancy < 2.3% with DTA < 0.3 mm in the build-up region	Discrepancies were within 2% in lung, 3% in light lung, up to 4.5% in air, 1.8% in bone, with slightly larger discrepancy (up to 5%) at interfaces	For 6 MV, average discrepancy of 1.1% in PDD and 1.6% in dose profiles. For 18 MV, average discrepancy of 1.6% in PDD and 3.0% and dose profiles	Discrepancies are mostly within 2%, with slightly higher discrepancy (up to 6%) at the air/tissue interface in the secondary build-up region	In general good agreement between AXB and MC, with an average gamma agreement with a 2%/1mm criteria of 91.3% to 96.8%

**Table 2:** A summary of information on some previous experimental validations for the accuracy of D-LBTE solvers in predicting the doses in heterogeneous humanoid phantoms using multiple clinical setup fields.

Published investigations	Han et al. 2012. [33]	Kan et al. 2013 [34]	Kan et al. 2012 [30]	Han et al. 2013 [35]	Hoffmann et al. 2012 [32]
Disease of interest	Oropharyngeal tumor	Nasopharyngeal carcinoma	Locally persistent nasopharyngeal carcinoma	Lung cancer	Tumor in mediastinum

Media involved	Water equivalent materials	Tissue, air, and bone	Tissue, air, and bone	Tissue and lung	Tissue, lung, and bone

Treatment technique used	IMRT, VMAT	IMRT, VMAT	IMSRT	IMRT, VMAT	A total of 11 different plans including opposing fields, multiple fields, IMRT, and VMAT.

Phantom used	RPC head and neck phantom	Anthropomorphic phantom (RANDO)	Anthropomorphic phantom (RANDO)	RPC thorax phantom	CIRS Thorax phantom

Measurement device	TLD and EBT film	TLD and EBT film	TLD	TLD and EBT film	EBT film

LBTE solver	AXB version 11 using both *D*_*m*_ and *D*_*w*_	AXB version 10 using both *D*_*m*_ and *D*_*w*_	AXB version 10 using *D*_*m*_ only	AXB version 11 using both *D*_*m*_ and *D*_*w*_	AXB version 10 using *D*_*m*_ only

Observed results	For TLD, deviation within 5%. For gamma analysis with film, 88% passed 5%/3 mm criterion for both *D*_*m*_ and *D*_*w*_	For TLD, deviation within 5%, with an average of 1.8%. For gamma analysis with film, 91% passed 3%/3 mm criterion for *D*_*m*_ and 99% for *D*_*w*_	For TLD, deviation within 3%	For TLD, deviation within 4.4%. For gamma analysis with film, ~97% passed 3%/3 mm criterion for *D*_*m*_ and 98% for *D*_*w*_	For gamma analysis with film, 98.2% passed the 3%/3 mm criterion for 6 MV and 99.5% for 15 MV
